# First Identification of MORF Family in Ferns: Molecular Regulation of Organellar RNA Editing in *Osmunda japonica* and *Plenasium vachellii*

**DOI:** 10.3390/biology14101463

**Published:** 2025-10-21

**Authors:** Lingling Li, Xiaolin Gu, Chuying Lu, Yingyi Liang, Jingyao Ping, Yingjuan Su, Ting Wang

**Affiliations:** 1 College of Life Sciences, South China Agricultural University, Guangzhou 510642, China; lilingling002020@163.com (L.L.); chuyinglu66@163.com (C.L.); 2 Shenzhen Branch, Guangdong Laboratory of Lingnan Modern Agriculture, Key Laboratory of Synthetic Biology, Ministry of Agriculture and Rural Affairs, Agricultural Genomics Institute at Shenzhen, Chinese Academy of Agricultural Sciences, Shenzhen 518120, China; guxl9523@163.com; 3 GMU-GIBH Joint School of Life Sciences, The Guangdong-Hong Kong-Macao Joint Laboratory for Cell Fate Regulation and Diseases, Guangzhou Medical University, Guangzhou 511436, China; yyliangchn@163.com; 4 Coconut Research Institute, Chinese Academy of Tropical Agricultural Sciences, Wenchang 571339, China; pingjnyao@foxmail.com; 5 School of Life Sciences, Sun Yat-sen University, Guangzhou 510275, China; 6 Research Institute of Sun Yat-sen University in Shenzhen, Shenzhen 518057, China

**Keywords:** RNA editing, MORF protein family, fern, Osmundaceae, organelle genome, transcriptomics

## Abstract

**Simple Summary:**

RNA editing critically regulates gene expression in plant organelles through post-transcriptional base substitution. We report the first identification of the Multiple Organellar RNA Editing Factor (MORF) family in ferns (*Osmunda japonica* and *Plenasium vachellii*). Through comparative transcriptomics and structural validation, we identified one MORF9 homolog in *O. japonica* and three homologs (MORF1/8/9) in *P. vachellii*, all containing conserved MORF-box domains. Our analysis demonstrates tissue- and organelle-specific RNA editing regulation: chloroplast editing frequencies are predicted to show dose-dependent enhancement (conserved sites 0.7–1; tissue-specific sites 0.1–0.2) potentially influenced by MORF presence, while mitochondrial editing exhibits uniform distribution. These findings challenge the traditional paradigm of MORF restriction to seed plants, revealing evolutionary conservation of RNA editing mechanisms in land plants.

**Abstract:**

RNA editing is a crucial mechanism regulating gene expression in plant organellar genomes, which optimizes protein structures through base substitution and plays a vital role in plant growth, development, and stress adaptation. This study revises the conventional understanding restricting MORF proteins to seed plants by reporting their first identification in ferns, an early vascular plant lineage. We sequenced chloroplast genomes of *O. japonica* and *P. vachellii*, revealing one MORF9 homolog in *O. japonica* and three homologs (MORF1/8/9) in *P. vachellii* through comparative transcriptomics and structural validation. All identified MORF proteins harbor conserved MORF-box domains, suggesting structural and potentially functional conservation with angiosperms. Crucially, MORF members differentially regulate organellar RNA editing: chloroplast editing frequencies are predicted to show dose-dependent enhancement (0.7–1.0 in conserved sites), potentially influenced by MORF presence or copy number. In *O. japonica*, chloroplast editing exhibits tissue-specific patterns (conserved sites 0.7–1.0; tissue-specific sites lower efficiency at 0.1–0.2), while this study’s mitochondrial editing results show a balanced frequency distribution (0–1 range). Amino acid substitution analysis demonstrates MORF-mediated hydrophobic optimization (Ser→Leu > 30%, Pro→Leu > 18%), likely underpinning fern adaptability. This work provides crucial initial evidence for a conserved MORF-mediated RNA editing module shared between these early vascular plants (ferns) and angiosperms, offering fundamental insights into the evolutionary trajectory of plant organellar gene regulation.

## 1. Introduction

RNA editing is hailed as the “hidden hero” in gene regulation. By altering the nucleotide sequence of RNA molecules, it breaks the direct link between genomic DNA and transcriptional products, thereby generating diversity and complexity in gene expression [1]. This unique mechanism not only influences gene functional performance but also provides a crucial entry point for investigating biological complexity. With the deepening understanding of RNA editing, we have gradually recognized its critical role in plant environmental adaptation and evolution. In 1986, Benne et al. made a groundbreaking discovery: uridine residues, which cannot be encoded by DNA, exist in the mitochondrial *cox*II gene transcript of trypanosomes. This finding revealed the previously unreported post-transcriptional regulatory mechanism of RNA editing [2]. In plants, RNA editing primarily occurs in mitochondria and chloroplasts. Through mechanisms such as C→U substitution, it corrects genomic mutations, optimizes protein structures, and plays a key role in regulating plant growth, development, and responses to environmental stress [3,4]. This enables plants to maintain growth advantages and adaptability in complex and changing environments. Since the first identification of C→U editing events in the mitochondria of *Oenothera biennis* in 1989, RNA editing in plant organelles has become a research focus, revealing its important role in plant life processes [5]. This progress has driven RNA editing research across multiple disciplines and broadened our understanding of plant biology. With further research, an ACG→ATG editing event was confirmed in the chloroplast rpl2 gene of maize (*Zea mays*), indicating that RNA editing mechanisms exist not only in model plants but also widely across plant species [6]. This discovery provides important evidence for further understanding the diversity of RNA editing in plants. Breakthroughs in high-throughput sequencing technology, particularly the application of DSN-seq, have significantly improved the detection sensitivity of low-abundance RNA. Combined with tools such as REDItools and RES-Scanner, researchers have successfully constructed genome-wide RNA editing maps for *Arabidopsis thaliana* and rice [7].

As systematic analysis of editing sites advances, the biological functions and molecular mechanisms of RNA editing have become research hotspots. RNA editing events regulate organelle function, plant growth and development, and abiotic stress responses through nuclear-encoded RNA editosomes (multicomponent protein complexes). These editosomes contain three core protein types: pentatricopeptide repeat (PPR) proteins, which recognize target RNA sequences [8]; DYW domain proteins, which catalyze deaminase reactions [9]; and the multiple organellar RNA editing factor (MORF, also called RIP) family, which serves as essential core components [10]. Among these, PPR proteins directly interact with mRNA to determine editing specificity, while MORF proteins participate in C→U editing through interactions with PPR proteins: PPR proteins recognize cytosine targets near editing sites, and MORF proteins regulate the RNA-binding activity of PPR proteins [11]. RNA editosome mutants typically exhibit developmental defects. Studies have shown that a knockout of MORF1, MORF3, or MORF8 reduces editing events [12], while in *A. thaliana*, chloroplast editing sites in MORF2 or MORF9 mutants reportedly lose nearly all function, indicating that MORFs have spatial specificity in RNA editing [13].

Traditional research suggests that MORFs constitute a small protein family in land plants. Since the first identification of MORF proteins in *A. thaliana* in 2012 [14], researchers have discovered homologous genes in various angiosperms, including monocots such as maize and rice, and dicots such as *Brassica napus*, *Populus trichocarpa*, and *Camellia sinensis* [15,16,17,18]. MORF proteins contain a conserved MORF-box domain and interact with PPR proteins by forming homodimers or multimers; however, their molecular functions remain unclear due to the lack of sequence similarity to known domains. Crystal structures of *A. thaliana* MORF1 and MORF9 reveal that both adopt a novel globular fold, confirming the multimerization mechanism of MORF proteins [19]. In terms of subcellular localization, MORF8 is localized to chloroplasts and mitochondria, MORF2 and MORF9 to chloroplasts, and the remaining members (MORF1, MORF3-7) to mitochondria [20]. MORF8 interacts with MORF1 in mitochondria and MORF2 in chloroplasts, specifically enhancing editing efficiency at sites such as *rpo*C-488 and *ndh*B-149, thereby promoting photosynthesis under low-temperature conditions [13]. These findings demonstrate that MORFs not only act as structural scaffolds but also regulate editosome stability, catalytic activity, and site specificity [21]. However, this knowledge is derived almost exclusively from angiosperms, leaving their presence and roles in earlier-diverging plant lineages largely unexplored.

Research on MORF proteins is well-advanced in angiosperms, but their presence in ferns—the oldest extant vascular plants—remains unreported [22]. Although the C→U RNA editing is widespread across land plants, with flowering plants exhibiting approximately 200–600 mitochondrial and 20–30 chloroplast editing sites, evidence from ferns remains limited. Limited studies on *Adiantum capillus-veneris* [23] and *Blechnopsis orientalis* [24] have reported editing events, primarily involving hydrophobic amino acid substitutions (e.g., Ser→Leu) that may influence organellar protein stability and membrane integration, akin to patterns in angiosperms.

Prior to this study, we conducted comprehensive bioinformatics analyses of publicly available fern genomic and transcriptomic datasets to identify potential MORF homologs. No definitive MORF-like sequences were detected, consistent with hypotheses that MORFs may be absent or rare in non-seed plants. This systematic preliminary effort provided a strong rationale for investigating whether MORF genes exist in ferns and have undergone distinct evolutionary divergence, addressing a key gap in understanding RNA editing mechanisms in early vascular plants.

Therefore, to begin to address this critical knowledge gap concerning fern MORF proteins, this study aimed to identify MORF proteins in the basal leptosporangiate fern family Osmundaceae, and integrated fern classification systems and phylogenetic relationships. Two fern species, *O. japonica* and *P. vachellii*, were selected since they can naturally hybridize to produce one critically endangered species *O. mildei* and hence are ideal materials to verify the MORF protein family. Here, we first sequenced chloroplast genomes of *O. japonica* and *P. vachellii*. We then successfully identified MORF proteins in *O. japonica* and *P. vachellii* using comparative analysis of transcriptome data with *A. thaliana* as a reference. Further analyses included phylogenetic evolution, homology comparisons, and RNA editing site identification based on lncRNA data combined with organelle genomes. Our results present the first direct evidence for the existence of MORF proteins in these specific fern species, thereby significantly advancing the traditional view where MORF proteins were thought to occur only in angiosperms and gymnosperms and not in lower vascular fern species. This study provides a critical initial foundation for in-depth research on the fern RNA editing system. 

## 2. Materials and Methods

### 2.1. Plant Material Collection and Sequencing

Fresh tissues of three Osmundaceae species were collected in July 2024 (Supplementary Figure S1): *O. japonica* (Baise, Guangxi: 105°48′5.5″ E, 24°39′18.0″ N, 1200 m above sea level), *P. banksiifolium* (Yanping, Fujian: 118°25′43″ E, 26°38’29″ N, 505 m above sea level), and *P. vachellii* (South China National Botanical Garden, Guangzhou: 113°23′51″ E, 23°11′34″ N). Healthy, pest-free individuals were selected with sampling strategies. For all three species (*O. japonica*, *Plenasium banksiifolium*, and *P. vachellii*), 1 fresh young leaf sample per species was collected for in-house chloroplast genome sequencing. For *O. japonica* and *P. vachellii*, additional root, sporophyll, and trophophyll samples were collected for conventional Illumina RNA-seq (3 biological replicates per tissue, 18 samples total) and lncRNA-seq with strand-specific library preparation (1 sample per tissue, 6 samples total). All samples were flash-frozen in liquid nitrogen and stored at −80 °C. Sequencing was performed on the Novaseg 6000 platform (llumina, San Diego, CA, USA) by Wuhan Benagen Technology Co., Ltd.(Wuhan, China).

### 2.2. Chloroplast Genome Assembly and Annotation

Chloroplast genome data from leaf tissues of *O. japonica*, *P. vachellii* and *P. banksiifolium* were assembled into circular sequences using GetOrganelle v1.7.5 with default parameters [25]. Genomes were annotated using CPGAVAS2 [26,27], and circular genome maps were visualized with OGDRAW [28]. tRNA genes were annotated using tRNAscan-SE [29], and rRNA genes were annotated via BLASTN [30]. Annotation errors in each chloroplast genome were manually corrected using CPGView [27] and Apollo [31]. The raw data of *O. japonica*, *P. vachellii*, and *P. banksiifolium* have been uploaded to the NCBI website, with the temporary accession project number PRJNA1321311.

### 2.3. Public Data Acquisition and Transcriptome Analysis

Transcriptome data of *Oryza sativa* ssp. *Japonica* Group (SRR2103721), *A. thaliana* MORF gene family sequences (At4g20020, At2g33430, At3g06790, At5g44780, At1g32580, At2g35240, At1g72530, At3g15000, At2g11430), and *O. sativa* ssp. *Japonica* Group genome data with annotation files were downloaded from the NCBI database(https://www.ncbi.nlm.nih.gov/#!/landingpage, accessed on 5 September 2024). Raw transcriptome sequencing data of *O. japonica* and *P. vachellii* (including 3 biological replicates) were also obtained. Identification of MORF family members was performed in two parts: (1) *O. sativa* ssp. *Japonica* Group MORF identification: A custom Hidden Markov Model (HMM) profile was first constructed using the hmmbuild program of HMMER v3.3.2 based on the nine *A. thaliana* MORF protein sequences as references. This HMM profile was then used to search the *O. sativa* ssp. *Japonica* Group genome-wide protein sequences [32] via HMMER v3.3.2 with an E-value ≤1 × 10^5^. Simultaneously, BLASTP 2.13.0 was used to align against *A. thaliana* reference sequences (E-value ≤ 1 × 10^5^). Candidate sequences were defined as the intersection of HMM and BLASTP results [33]. Subsequently, MEME analysis [34] (default parameters, 3 motifs) was performed to characterize the motif architecture of these candidates and confirm the presence of the conserved MORF-box domain. Sequences were verified based on the conserved MORF-box domain, defined by its sequence composition, key conserved residues, relative motif order, motif length > 20, and E-value ≤ 1 × 10^5^, with complete motif sets. MEME motif numbering (e.g., motif2-motif3-motif1 in our specific MEME run) was used solely for visualization of motif arrangement patterns and not as a filtering criterion. These stringent steps yielded the final *O. sativa* ssp. *Japonica* Group MORF family members. (2) *O. japonica* and *P. vachellii*. MORF identification: Raw transcriptome sequencing data were quality-filtered to remove adapters and low-quality reads. De novo transcriptome assembly was performed using Trinity v2.15.2 [35], followed by redundancy reduction with CD-HIT v4.8.1 [36]. Coding sequences (CDS) and corresponding protein sequences were predicted via TransDecoder v5.5.0 [37]. Predicted proteins were aligned to *A. thaliana* MORF references using BLASTP (E-value ≤ 1 × 10^5^), combined with HMMER v3.3.2 searches (E-value ≤ 1 × 10^5^) using the same custom HMM profile constructed from *A. thaliana* MORF protein sequences as described in (1) for *O. sativa* ssp. *Japonica* Group. MEME motif analysis was performed with the same criteria as in (1), confirming the conserved MORF-box domain based on sequence composition, key conserved residues, relative motif order, motif length > 20, and E-value ≤ 1 × 10^5^. MEME motif numbering was used solely for visualization purposes and not as a filtering criterion. Final MORF members were confirmed by verifying the conserved MORF-box domain and named using the “species abbreviation + number” convention.

### 2.4. Analysis of Protein Structure and Physicochemical Properties

To systematically characterize the MORF protein families in *A. thaliana*, *O. sativa* ssp. *Japonica* Group, *O. japonica*, and *P. vachellii*, their physicochemical properties were first predicted using the ExPASy ProtParam online tool (https://web.expasy.org/protparam, accessed on 5 January 2025), including total amino acid residues (AA), molecular weight (MW, unit: Da), theoretical isoelectric point (pI), instability index (II; ≤40 indicates stable protein), aliphatic index (AI), and grand average of hydropathicity (GRAVY; negative values indicate hydrophilicity). Multiple sequence alignment was performed using MEGA11 (v11.0.13) [38], Secondary structure elements (α-helices, β-sheets, and random coils) were predicted via the ESPript 3.0 online tool (https://espript.ibcp.fr/ESPript/cgi-bin/ESPript.cgi, accessed on 8 January 2025), and the same tool was used to optimize sequence visualization (e.g., highlighting conserved sites) and annotate the predicted secondary structures onto the alignment (α-helices as orange lines, β-sheets as blue arrows), thereby visually illustrating spatial correlations between conserved domains and secondary structures.

For the predicted members of the MORF protein family in *O. japonica* and *P. vachellii*, further tertiary structure modeling and comparative analysis were conducted. The identified target protein sequences were submitted to the SWISS-MODEL server (https://swissmodel.expasy.org/, accessed on 2 February 2025). With the NCBI Structure database as the template source, templates were screened based on the criteria of high amino acid homology, high software scores, and wide coverage, and files in PDB format were output. Subsequently, the PDB files were visualized using PYMOL (v2.5.2) software. Meanwhile, the 3 conserved motifs (Motif 1–3) predicted by MEME Suite were labeled with red, yellow, and blue colors in the 3D models, which intuitively showed the conservation of motif topological structures in MORF proteins from different species.

### 2.5. Prediction of RNA Editing Sites in Organelle Genomes Across Multiple Plant Groups

RNA editing sites in 26 chloroplast and 21 mitochondrial genomes were predicted using the PREPACT3 online tool (http://www.prepact.de/prepact-main.php, accessed on 8 February 2025) to investigate differences in organelle genome editing patterns across plant lineages. The chloroplast genome dataset included 26 species from four major plant lineages traditionally recognized in land plant classification: 1 bryophyte (*Anthoceros punctatus*); 13 ferns (*Todea barbara*, *Claytosmunda claytoniana*, *P. vachellii*, *P. banksiifolium*, *Plenasium angustifolium*, *O. japonica*, *Osmunda mildei*, *Osmunda javanica*, *Dryopteris crassirhizoma*, *Blechnopsis orientalis*, *Psilotum nudum*, *Ophioglossum vulgatum*, *Ophioglossum californicum*); 4 gymnosperms (*Welwitschia mirabilis*, *Pinus taeda*, *Cycas taitungensis*, *Ginkgo biloba*); 4 monocot angiosperms (*Oryza sativa Indica* Group, *Triticum aestivum*, *Phoenix dactylifera*, *Cocos nucifera*); and 4 dicot angiosperms (*Liriodendron tulipifera*, *Aconitum kusnezoffii*, *Nelumbo nucifera*, *A. thaliana*). The mitochondrial genome dataset included 19 species overlapping with the chloroplast dataset (spanning the 4 lineages above). Default software thresholds were used for prediction, with outputs including C→U editing site positions and amino acid substitution types (e.g., Ser→Leu) before and after editing. Editing characteristics across plant lineages were analyzed by quantifying editing site counts, C→U type proportions, and amino acid substitution preferences (e.g., frequency of hydrophobic amino acid replacements). Special emphasis was placed on differentiating editing features between Osmundaceae species and other lineages. Additionally, differences in editing patterns were explored between organelle genomes of species with MORF protein family members and those without.

### 2.6. Identification of Tissue-Specific RNA Editing Sites in Osmundaceae Species

To investigate interspecific divergence and intraspecific tissue specificity of organelle genome RNA editing in Osmundaceae, this study integrated multi-omics data for systematic analysis: lncRNA-seq data from three tissues (vegetative leaves, sporophylls, roots) of *O. japonica* and *P. vachellii* were generated in-house (see Section 2.1 for details); for organelle genomes: *O. japonica* chloroplast genome was sequenced in this study (Section 2.3), and its mitochondrial genome was retrieved from NCBI (accession: PQ202832); *P. vachellii* analysis only included the chloroplast genome sequenced herein (mitochondrial genome data unavailable); transcriptome data of *P. banksiifolium* were obtained from the NCBI SRA database (accession: SRR6920617) for interspecific comparative analysis. We adopted a two-step strategy to predict RNA editing sites. First, IncRNA-seq reads were mapped to the coding sequences (CDS) of each protein-coding gene (PCG) using BWA (v0.7.17) with default parameters. Subsequently, RNA editing sites were predicted using REDItools based on the mapping results, with the following criteria: coverage > 30, frequency ≥ 0.1 [39]. This *p*-value threshold was set to broadly capture all potential RNA-DNA sequence discrepancies, including both true RNA editing events and genomic single nucleotide polymorphisms (SNPs), for subsequent rigorous exclusion. Next, Illumina DNA short reads were aligned to the CDS of each PCG using BWA software with default parameters. Genomic SNPs were then predicted using BCFtools based on these alignment results with thresholds set as coverage >30 and frequency ≤0.1 [40]. These naturally occurring polymorphic sites (i.e., SNPs) need to be excluded from the candidate RNA editing sites. Finally, after removing SNP sites, the remaining sites identified from the IncRNA-seq mapping results were regarded as authentic RNA editing sites.

### 2.7. Statistical Analysis

Quantitative data from RNA editing site prediction (Section 2.5 and Section 2.6) and other derived metrics were statistically analyzed using R (v4.2.2), an open-source statistical computing environment [41]. To rigorously analyze correlations among different amino acid substitution types resulting from RNA editing and to address the intrinsic issues of compositional data (where the sum of proportions is constant, potentially leading to spurious negative correlations), log-ratio transformations were employed. Specifically, the relative frequency of each amino acid substitution type was first calculated for each biological sample. These proportional data were then transformed using the Centered Log-ratio (CLR) transformation. This transformation maps the compositional data from the simplex into a Euclidean space, effectively removing the constant-sum constraint and thereby enabling the application of standard statistical analyses. Following CLR transformation, Pearson correlation coefficients were calculated between the transformed frequencies of different amino acid substitution types. This methodological approach ensures that any observed positive or negative correlations are more likely to reflect genuine biological interactions (synergy or antagonism) rather than mathematical artifacts.

## 3. Results

### 3.1. Identification and Characterization of MORF Proteins in O. japonica and P. vachellii

MORF family members were identified using a combined approach of Hidden Markov Model (HMM) and BLASTP alignment, utilizing genomic data from *O. sativa* ssp. *Japonica* Group and transcriptomic data from *O. japonica* and *P. vachellii*. After redundancy removal via CD-HIT, online HMM validation (E-value ≤ 1 × 10^5^), and MEME conserved motif analysis (confirming the presence and consistent arrangement of the complete motif2-motif3-motif1 sequence), we identified 7 MORF members in rice, 1 in *O. japonica* (OJ_27007), and 3 in *P. vachellii* (PV_23919, PV_33438, PV_35689). All members contain a conserved MORF-box domain, consistent with findings in *A. thaliana*, rice, maize, and other species.

**Figure 1:**
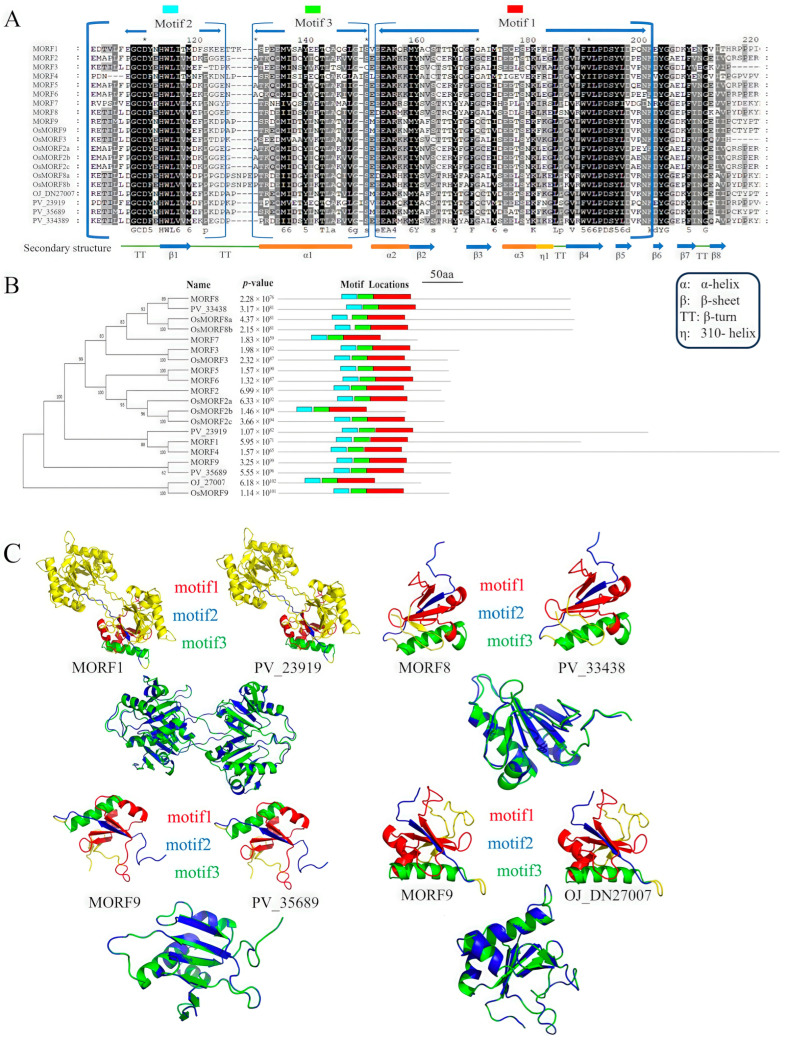
Structural and Evolutionary Analysis of the MORF Protein Family: (**A**) Multiple sequence alignment of 20 MORFs proteins (*A. thaliana*, *O. sativa* ssp. *Japonica* Group, *O. japonica*, *P. vachellii*), highlighting the conserved ~100-amino-acid MORF box. Secondary structure elements (α-helices, β-strands, etc.) and conserved residues (60%, inverse shading), and colored motifs (red: Motif 1, blue: Motif 2, green: Motif 3) are annotated. (**B**) Left: Phylogenetic tree of MORF evolutionary relationships. Right: Conserved motif (Motif 1–3) distribution aligned with the tree (red/blue/green as in A), with protein names, *p*-values, and motif positions summarized. (**C**) 3D structural comparisons of *A. thaliana* MORFs with *O. japonica*/*P. vachellii* homologs (motifs colored red/blue/green as in A); nested arrangement highlights structural conservation.

Phylogenetic tree construction using the Maximum Likelihood (ML) method revealed that OJ_27007 from *O. japonica* clustered with MORF9 from *A. thaliana* (Figure 1B). Additionally, PV_23919, PV_35689, and PV_33438 from *P. vachellii* clustered with MORF1, MORF8, and MORF9 from *A. thaliana*, respectively. Secondary structure analysis indicated that the three motif regions contain three conserved α-helices (α1–α3) and five β-sheets (β1–β5). Notably, α-helices and β-sheets accounted for the highest proportion and were highly conserved (Figure 1A).

Analysis using the Expasy-ProtParam online tool revealed the following properties of MORF proteins (Table 1). Molecular weights ranged from 19,720.09 Da (OsMORF2b) to 78,822.18 Da (AtMORF4), with a positive correlation between amino acid number and molecular weight. Theoretical isoelectric points (pI) varied from 5.64 (OsMORF2b, acidic) to 9.38 (AtMORF8, basic), with most proteins being weakly basic (pI > 7). Instability indices were ranged from 44.76 (AtMORF4) to 79.79 (AtMORF7), classifying all as unstable proteins (instability index > 40). Aliphatic indices (43.29 for AtMORF4 to 79.79 for AtMORF7) were differences in thermal stability among members; and grand average of hydropathicity (GRAVY) values were all negative (−1.001 to −0.476), confirming all MORF proteins are hydrophilic with no detected transmembrane domains.

Tertiary structure prediction of *O. japonica* OJ_27007 and *P. vachellii* PV_23919, PV_33438, and PV_35689 was performed using the SWISS-MODEL server, with *A. thaliana* MORF proteins as templates. PYMOL visualization (Figure 1C) showed that the spatial conformations of *O. japonica* OJ_27007 and *P. vachellii* PV_35689 were highly consistent with that of *A. thaliana* MORF9 (RMSD = 1.2 Å). The conserved motifs predicted by MEME (Motif1: red, Motif2: yellow, Motif3: blue) were mainly distributed in α-helix or β-sheet regions, and their spatial positions were highly conserved among homologous proteins from different species, providing structural biological evidence for the functional conservation of the MORF family in ferns.

### 3.2. Comparative Analysis of Organellar RNA Editing Across Plant Taxa

**Figure 2:**
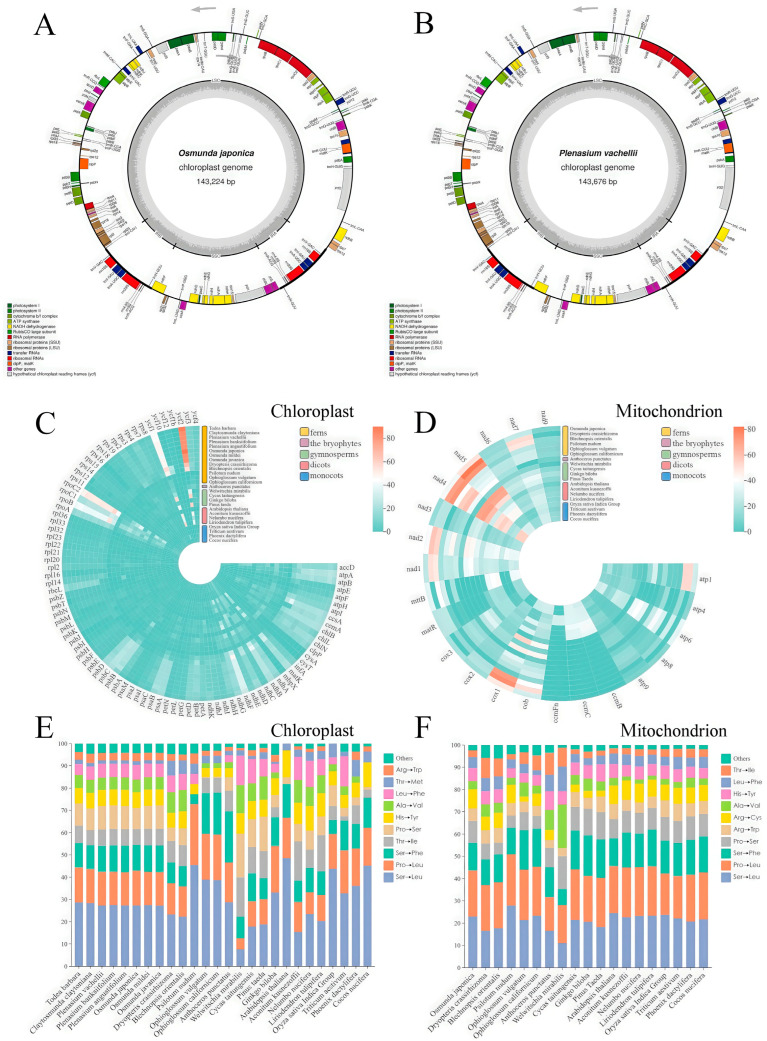
Organellar genome structure and RNA editing landscapes in Osmundaceae and other plant lineages: (**A**) *O. japonica* chloroplast genome. Transcription direction: Genes located outside the outer circle are transcribed in a counterclockwise direction, while genes located inside the outer circle are transcribed in a clockwise direction. (**B**) *P. vachellii* chloroplast genome. Transcription direction follows the same convention as in (**A**): Genes outside the outer circle are transcribed counterclockwise, and genes inside are transcribed clockwise. (**C**) Circular heatmap of chloroplast RNA editing sites (26 species). The outer ring denotes gene names, and the inner ring radius corresponds to species names, ordered taxonomically from top to bottom: ferns, bryophytes, gymnosperms, dicots, and monocots. Color intensity reflects the abundance of editing sites. (**D**) Circular heatmap of mitochondrial RNA editing sites (19 species), with taxonomic ordering and annotations as in (**C**). (**E**) Stacked bar plot showing the frequency distribution of amino acid change types in chloroplast genomes across 26 species. Different colors represent distinct amino acid editing types. (**F**) Stacked bar plot of mitochondrial amino acid editing type frequencies in 19 species, following the same color-coding for amino acid change types as (**E**).

To bolster genomic resources in Osmundaceae and facilitate broad comparisons of RNA editing across plant lineages, we sequenced and compared the chloroplast genomes of *O. japonica* and *P. vachellii*. Both genomes possess the typical quadripartite structure of land plants (Figure 2A,B) and exhibit high consistency in key features such as genome size (143,224 bp vs. 143,676 bp), GC content (40.51% vs. 40.41%), and gene composition (both containing 85 protein-coding genes, 33 tRNA genes, and 4 rRNA genes) (Supplementary Table S1). These high similarities in genome structure, base composition, and gene content strongly support a close evolutionary relationship between *O. japonica* and *P. vachellii*.

To characterize predicted RNA editing patterns across diverse plant lineages, we compared predicted editing sites in chloroplasts and mitochondria of bryophytes, ferns, gymnosperms, and angiosperms using PREPACT. The analysis revealed that predicted RNA editing events are significantly unevenly distributed among plant lineages. In chloroplasts (Figure 2C), ferns exhibit far higher editing activity (inferred from predicted sites) than other groups, with *Dryopteris crassirhizoma* and *Claytosmunda claytoniana* both having over 900 predicted editing sites. Notably, Osmundaceae species consistently have a high number of predicted editing sites, ranging from 880 to 930, which is significantly higher than other lineages (*p* < 0.0001). This may be attributed to their conserved genome structure and shared evolutionary pressures. Functionally, predicted editing sites are putatively highly enriched in key genes such as NADH dehydrogenase subunit (*ndh*B), chloroplast membrane proteins (*ycf*1/*ycf*2), and transcription-related (*rpo*) genes, highlighting the putative critical role of RNA editing in ensuring the stable performance of core biological functions. RNA editing in mitochondria also shows strong lineage specificity based on predicted sites (Figure 2D), with ferns once again representing the group with the most frequent predicted editing activity. Compared with other lineages, species of Osmundaceae exhibit a unique editing signature: their *ndh*5 gene, which encodes NADH dehydrogenase subunit 5, contains significantly more predicted editing sites than those of other ferns (*p* < 0.05), a feature that may enhance the plasticity of electron transfer in the mitochondrial respiratory chain and contribute to environmental adaptability. In contrast, the *ccm*B gene, which encodes cytochrome c maturation protein B—a crucial component in the pathway for assembling functional cytochrome c in the mitochondrial electron transport chain—has significantly fewer predicted editing sites (*p* < 0.01), reflecting high conservation in its growth regulatory function. Regarding the editing outcomes, a general trend is that predicted RNA editing tends to convert amino acid codons to those encoding more hydrophobic amino acids. Among these, the amino acid substitution patterns induced by predicted editing are relatively conserved in the chloroplasts of ferns (Figure 2E), while showing higher consistency across a broader range of plant lineages in mitochondria (Figure 2F).

### 3.3. Interplay Among RNA Editing-Induced Amino Acid Changes Across Plant Taxa

**Figure 3:**
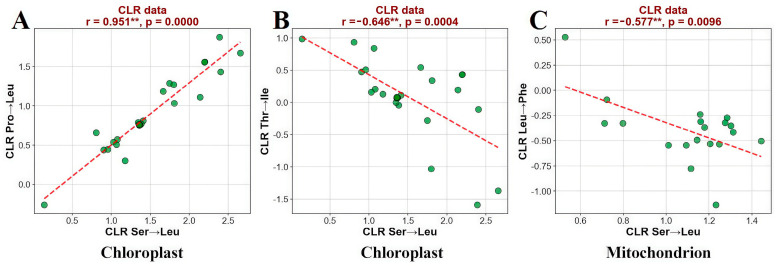
Frequency analysis of amino acid change types in chloroplast and mitochondrial genomes across multiple species (** represents significant at the 0.01 level): (**A**). Correlation analysis between Ser→Leu and Pro→Leu. (**B**). Correlation analysis between Ser→Leu and Thr→Ile. (**C**). Correlation analysis between Ser→Leu and Leu→Phe.

To investigate the patterns of RNA editing-induced amino acid changes, this study conducted statistical analyses on chloroplast gene editing sites across 26 plant taxa. Results revealed that Ser→Leu (serine→leucine) and Pro→Leu (proline→leucine) were the dominant editing types, both reaching extremely significant levels (*p* < 0.001). Prior to correlation analysis, proportional data were subjected to Centered Log-ratio (CLR) transformation to address inherent issues associated with compositional data (i.e., constant sum of proportions potentially leading to spurious negative correlations). Further correlation analysis indicated a significant positive association between these two amino acid conversion types (*p* < 0.01, Figure 3A). A shared characteristic is their generation of hydrophobic leucine residues, suggesting this process may influence molecular function by enhancing the folding stability of the protein hydrophobic core. Additionally, a highly significant negative correlation was observed between Ser→Leu and Thr→Ile (threonine→isoleucine) editing types (*p* < 0.01, Figure 3B), implying that different editing types may exert antagonistic effects by competing for limited editing complexes or recognition elements. Analysis of mitochondrial gene editing sites in 19 plant taxa showed that Ser→Leu, Pro→Leu, and Ser→Phe (serine→phenylalanine) were the predominant conversion types, with an overall distribution pattern highly consistent with that of chloroplasts (Figure 3C). Notably, a highly significant negative correlation was found between Ser→Leu and Leu→Phe (leucine→phenylalanine) editing in mitochondria (*p* < 0.01), suggesting these two editing events may be governed by mutually exclusive regulatory mechanisms. For instance, editing priority allocation could be achieved through specific binding to different PPR proteins or dependence on the sequence context characteristics of editing sites.

### 3.4. Tissue- and Organelle-Specific RNA Editing Sites in O. japonica and P. vachellii

**Figure 4:**
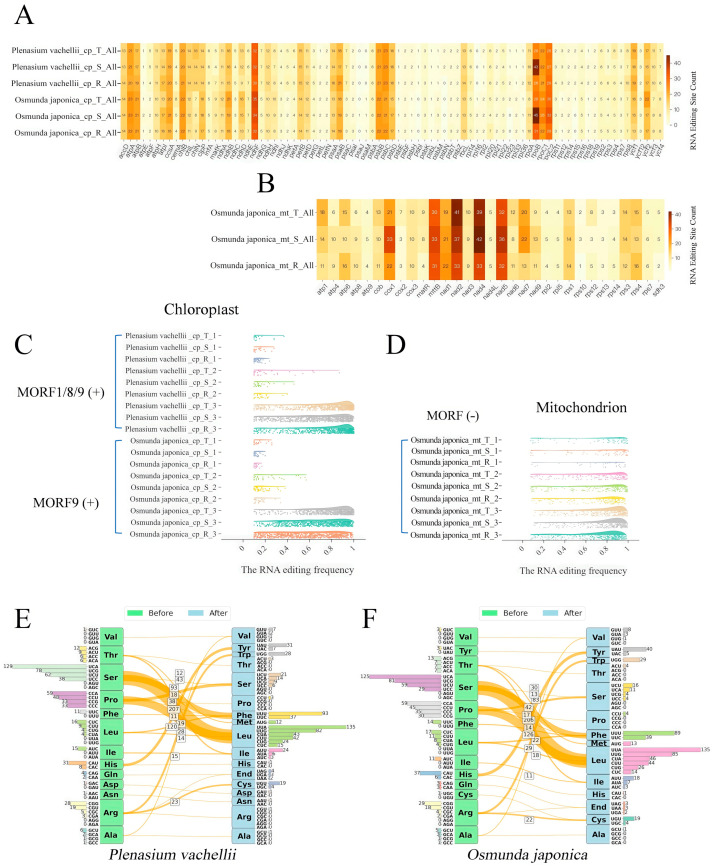
RNA editing site profiles in organelle genomes across tissues of *O. japonica* and *P. vachellii* (**A**) Grid plot displaying the number of RNA editing sites per chloroplast gene across six samples, with numerical values in individual cells representing editing site counts for each gene; darker colors indicate higher numbers of RNA editing sites. Samples include three tissues (trophophyll [T], sporophyll [S], and root [R]) from two species. (**B**) Grid plot displaying the number of RNA editing sites per chloroplast gene across three samples, with numerical values in individual cells representing editing site counts for each gene; darker colors indicate higher numbers of RNA editing sites. Samples include three tissues (trophophyll [T], sporophyll [S], and root [R]) from *O. japonica*. (**C**) The cloud plot shows the RNA editing frequencies of tissue-specific editing sites (T_1/S_1/R_1), cross-tissue sites (T_2/S_2/R_2), and tri-tissue overlapping sites (T_3/S_3/R_3) filtered from the chloroplast genomes of *P. vachellii* and *O. japonica*. (**D**) The cloud plot shows the RNA editing frequencies of tissue-specific editing sites (T_1/S_1/R_1), cross-tissue sites (T_2/S_2/R_2), and tri-tissue overlapping sites (T_3/S_3/R_3) filtered from the mitochondrion genomes of *O. japonica*. (**E**) Amino acid changes and codon frequencies before and after RNA editing in *P. vachellii* (**F**) Amino acid changes and codon frequencies before and after RNA editing in *O. japonica*.

In different tissues (vegetative leaves, sporophylls, roots) of *O. japonica* and *P. vachellii*, we identified approximately 700 high-confidence C→U RNA editing sites within 85 chloroplast protein-coding genes. The editing patterns showed high tissue conservation within homologous genes (Figure 4A). Genes with high editing frequencies were mainly enriched in energy metabolism-related gene families (*atp*A, *atp*B, *ndh*F) and transcription-related gene families (*rpo*B, *rpo*C). A total of 471 conserved editing sites (67.3% of the total) were shared across tissues between the two species, with 153 species-specific conserved sites in *O. japonica* and 146 in *P. vachellii*. The number of tissue-specific sites was small (Supplementary Figure S2). Notably, the overall chloroplast RNA editing frequency in *P. vachellii* was higher than in *O. japonica*. This observation may be tentatively hypothesized to be related to the differing numbers of identified MORF protein family members: *P. vachellii* contains 3 putative MORF proteins (MORF1, MORF8, MORF9), while only 1 MORF9 homolog was identified in *O. japonica*, and this predicted protein is localized in chloroplasts (based on subcellular localization prediction). Building on findings from angiosperms, it is plausible that a higher number of MORF proteins might facilitate more extensive protein interactions, potentially enhancing the stability or activity of the editing complex, and thereby contributing to improved overall editing efficiency. However, direct experimental evidence is required to confirm this causal link in ferns. Further analysis showed that both species exhibited the characteristic that “editing efficiency of tissue-specific sites (0.1–0.2) was significantly lower than that of conserved sites (1.0)”, but the editing frequency of specific sites in *P. vachellii* was still slightly higher than in *O. japonica* (Figure 4C), providing correlative evidence that is consistent with the hypothesis of a positive regulatory role for the number of MORF proteins on editing efficiency, though causality requires experimental verification. In the 31 mitochondrial genes of *O. japonica*, approximately 500 high-confidence C→U editing sites were identified in the three tissues, which was 30% less than that in chloroplasts (Figure 4B). Similarly to chloroplasts, the number of editing sites in mitochondrial homologous genes was also highly conserved among tissues. High-frequency editing sites were concentrated in respiratory chain-related genes (*cox*1, *nad*2, *nad*4, *nad*5) and ATP synthase-related genes (*atp* family), and the editing efficiency of conserved sites was nearly complete (frequency ~1.0) (Figure 4D). Cross-tissue analysis showed that 292 conserved sites were shared among all tissues, with a small number of tissue-specific sites (60 in vegetative leaves, 53 in sporophylls, and 34 in roots). Unlike chloroplasts, the editing frequency distribution of tissue-specific sites in *O. japonica* mitochondria was more uniform. This could potentially be related to the apparent absence of MORF9 homologs in its mitochondria based on our current identification method. We hypothesize that if MORF protein regulation is indeed absent in mitochondria, the mitochondrial editing process might rely on other basic editing factors, potentially leading to a more balanced distribution of editing efficiency between conserved and specific sites. This hypothesis warrants further investigation into the presence, localization, and function of MORF proteins and other editing factors in fern mitochondria. Amino acid substitution analysis showed that both species had strong preference patterns: Ser→Leu (30%), Pro→Leu (18%), Ser→Phe (12%), and Pro→Ser (5%) accounted for 65.0% of total substitutions (Figure 4E,F), and the hydrophobicity of proteins was significantly enhanced after substitution (*p* < 0.01), supporting the “hydrophobic core stabilization” hypothesis. The high similarity in editing patterns and amino acid substitution profiles may reflect functional constraints of closely related species under shared ecological niches.

### 3.5. Highly Conserved Chloroplast RNA Editing Sites of O. japonica, P. vachellii and P. banksiifolium

**Figure 5:**
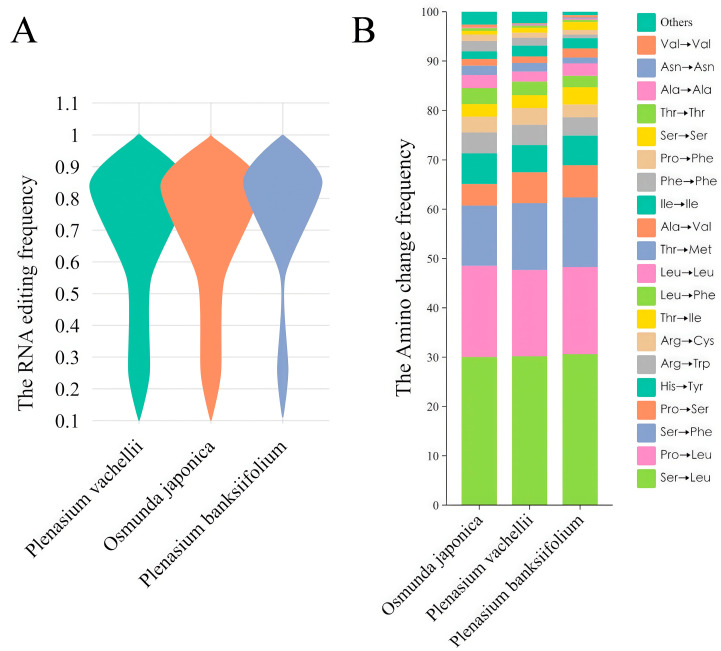
RNA editing site profiles among *O. japonica*, *P. vachellii*, and *P. banksiifolium:* (**A**) Violin plot showing the distribution of RNA editing frequencies in chloroplast genomes of leaf tissues across the three species. (**B**) Bar plot displaying the types and frequencies of amino acid changes before and after RNA editing in leaf chloroplast genomes, with relative frequencies summing to 100% on the *y*-axis.

Transcriptome analysis of *P. banksiifolium* identified no putative MORF protein family members within the scope of our current identification method and available data. Using its chloroplast genome data, we annotated chloroplast RNA editing sites and compared them across three fern species: *O. japonica* (1 MORF), *P. vachellii* (3 MORFs), and *P. banksiifolium* (0 MORFs). Comparative analysis (Figure 5A) revealed minimal differences in editing site counts and highly similar editing frequency distributions across species, with frequencies predominantly clustering between 0.7–0.9. Statistical analysis of amino acid substitutions induced by editing identified five dominant types, collectively accounting for >60% of all edits (Figure 5B): Ser→Leu (30%), Pro→Leu (18%), Ser→Phe (13%), Pro→Ser (6%), and His→Tyr (5%). These results indicate that chloroplast genomes in the leaf tissues of the three species exhibit high conservation in overall editing patterns. This presents an interesting observation regarding the potential independence of basal editing levels from MORF presence in certain contexts, an aspect that warrants further discussion.

## 4. Discussion

As a key mechanism regulating gene expression in plant organelles, RNA editing plays a central role in the adaptive evolution of species [42,43]. By precisely regulating gene expression patterns, this mechanism enables plants to rapidly adjust metabolic networks and physiological activities under environmental stress, thereby significantly enhancing their environmental adaptability and survival competitiveness. Notably, the regulatory effects of RNA editing are not only manifested at the protein translation level [44], but also exert profound regulation on plant growth and development processes as well as environmental response strategies by systematically influencing the expression profiles and functional networks of organelle genes [4,43]. While functional studies of RNA editing have made significant progress in angiosperms, research on related mechanisms in ferns—a group of early vascular plants—remains in the exploratory stage [22]. In particular, there have been no systematic reports on the MORF protein family, a core component of the RNA editosome in ferns. In fact, due to the abundance of RNA editing sites and diverse editing types in their organelle genomes, ferns should have been ideal materials for analyzing the molecular mechanisms and adaptive evolutionary patterns of organelle RNA editing. Prior to this study, the MORF protein family, a core component of the RNA editosome, had not been systematically identified in ferns [22]. This gap likely stems from the high complexity and large size of fern genomes, which may hinder whole-genome sequencing. Consequently, limited public genomic data for ferns may restrict investigations of RNA editing factors, such as MORF proteins. In contrast, abundant genomic resources for seed plants, particularly angiosperms, have enabled extensive organellar RNA editing studies. This disparity highlights the value of our study, which provides initial bioinformatics evidence of MORF proteins in ferns, potentially contributing to understanding RNA editing in early vascular plants, pending experimental validation.

As highlighted in the Introduction, prior extensive searches in publicly available fern genomic and transcriptomic data had not yielded systematic identification of MORF protein family members. This study provides initial evidence of RNA editosome proteins, specifically members of the MORF protein family, in the fern species *O. japonica* and *P. vachellii*. As a primitive group of ferns, Osmundaceae plants exhibit strong adaptability and abundant RNA editing sites [45]. This finding contributes to filling the previous gap in documented evidence for MORF proteins in ferns [22], offering valuable insights into RNA editing mechanisms in this early vascular plant group. Our research shows that *O. japonica* and *P. vachellii* not only possess highly conserved RNA editing sites but also have far more editing sites in chloroplast genes than angiosperms. This elevated editing site count likely reflects retention of an ancestral state in early-diverging land plants, progressively lost in seed plants via accelerated C→T substitutions that fix edited cytidines genomically, reducing post-transcriptional editing needs [46]. Additionally, the energetic costs of sustaining extensive RNA editing machinery, including large PPR and MORF families, may have favored loss of superfluous sites in angiosperms, promoting genomic streamlining [47]. Specifically, approximately 700 C→U editing sites were identified in vegetative leaves, sporophylls, and roots, with significant variation in the number of editing sites among different genes. Furthermore, RNA editing frequency varies across different tissues. In particular, editing patterns in vegetative leaves, sporophylls, and roots exhibit tissue-specific characteristics of RNA editing “activation.” This result suggests that the activation of RNA editing may be subject to tissue-specific regulation. Drawing on studies in angiosperms, this regulation may be hypothesized to result from differential expression of editing factors, including PPR and MORF proteins, potentially modulated by developmental and environmental cues [48]. Similarly, organelle-specific editing in ferns may be hypothesized to be influenced by the selective localization of these proteins to chloroplasts or mitochondria, coupled with distinct RNA environments in each organelle, though further fern-specific studies are needed to confirm these mechanisms.

In the chloroplast genome, the average editing frequency of *P. vachellii* is higher than that of *O. japonica*, which in turn is higher than that of fern species where no MORF proteins have been identified. Three MORF protein family members (MORF1, MORF8, MORF9) were identified in *P. vachellii*, whereas only MORF9 was found in *O. japonica*. Previous studies have shown that MORF proteins enhance editing efficiency by assisting PPR proteins through protein–protein interactions and can form homodimers and heterodimers among themselves [49]. For instance, in maize, ZmMORF1 and ZmMORF8 strengthen the synergistic effect between EMP7 and PCW1, enabling efficient editing [12]. These findings suggest that the number of MORF protein members and their interactions may play key roles in facilitating RNA editing in the chloroplast genome. Based on these observations and existing angiosperm data, it is hypothesized that the presence and interaction of MORF proteins may contribute to enhancing RNA editing efficiency, thereby potentially promoting normal plant growth, development, and adaptability. This hypothesis is consistent with similar findings in angiosperms [50].

However, findings for *P. banksiifolium* (where no MORF proteins were identified) indicate that editing patterns are comparable to *O. japonica* and *P. vachellii*, suggesting no simple relationship with MORF presence. This may reflect limitations in the *P. banksiifolium* transcriptome data from NCBI SRA, including lower sequencing depth and coverage compared to our high-depth datasets, potentially obscuring MORF detection or editing precision. Other factors, such as core RNA editing machinery, may also maintain baseline editing levels independently of MORF proteins. This underscores the complexity of RNA editing regulation in ferns and the need for deeper sequencing and functional studies.

In addition, we compared RNA editing patterns between the chloroplast and mitochondrial genomes of *O. japonica*. Our analysis revealed significant differences in editing frequencies between conserved and tissue-specific editing sites in the *O. japonica*. chloroplast genome: conserved sites showed generally high editing frequencies (0.7–1.0), whereas tissue-specific sites typically had frequencies below 0.3. In the mitochondrial genome, however, there was little difference in editing frequencies between these site types, with a balanced distribution (0–1 range). This phenomenon was not accidental, as similar results were observed across three tissue types in *O. japonica*. This pattern may be influenced by the absence of mitochondrial-localized MORF proteins, as only a MORF9 homolog (predicted to localize to chloroplasts, based on angiosperm data) was identified [51]. Thus, mitochondrial editing in this study appears unaffected by MORF-specific modulation, remaining balanced, though experimental validation of localization and function in ferns is required.

We further investigated how RNA editing affects the types of amino acid changes in proteins. In *O. japonica* and *P. vachellii*, we found that amino acid changes at RNA editing sites primarily tend to produce hydrophobic amino acids. This is consistent with RNA editing patterns in angiosperms, where enhanced protein hydrophobicity helps maintain organelle function-particularly the structure of photosynthesis-related complexes [52,53]. By comparing RNA editing patterns across different plant groups, we observed that fern chloroplast genomes generally contain a larger number of RNA editing sites, and these sites are relatively conserved. Notably, the number of RNA editing sites shows high conservation among several species within the Osmundaceae family. Furthermore, we observed that amino acid changes at RNA editing sites—both in chloroplasts and mitochondria—primarily involve the production of hydrophobic amino acids. This result suggests that RNA editing in organelle genomes might share functional commonality, which could contribute to maintaining protein stability.

Furthermore, we found that there are positive and negative correlations between amino acid type changes before and after RNA editing. Our research reveals the universality of this phenomenon across multiple plant groups, particularly the consistent correlation patterns of RNA editing types in organelle genomes of different plant taxa [54]. In our study, the frequency of Ser→Leu editing showed a significant positive correlation with that of Pro→Leu editing in the chloroplast genomes of five different plant groups, whereas the frequency of Ser→Leu editing was significantly negatively correlated with that of Thr→Ile editing. In mitochondrial genomes, a significant negative correlation was observed between the frequencies of Ser→Leu and Leu→Phe editing types. These findings tentatively suggest that RNA editing mechanisms might have diverged between organelles, characterized by potential synergistic or antagonistic interactions among RNA editing types. They also imply that repair and diversification mechanisms in plant RNA editing can confer adaptive evolutionary advantages [25,55].

These new findings provide initial bioinformatics insights for future studies on organelle gene regulation, particularly regarding RNA editing patterns and amino acid changes. We hypothesize that interrelationships among editing types may reflect organelle-specific mechanisms, pending further validation. This study offers preliminary perspectives on RNA editing in ferns. Nevertheless, this research has several limitations. Our observations on MORF presence and editing frequencies are speculative, based on bioinformatics correlations and angiosperm extrapolations, with no direct causal evidence; *P. banksiifolium* data limitations (e.g., low-depth sequencing) further highlight the need for improved datasets. Synergistic MORF mechanisms require experimental validation (e.g., Co-IP). Spatiotemporal MORF expression needs clarification via advanced transcriptomics. Amino acid impacts on adaptation require mutant and metabolomic studies.

Although this study provides important evidence for RNA editing mechanisms in ferns, future research should further explore the functions and evolution of MORF proteins across different plant groups, particularly their diversity and functional differences in non-model plants. Furthermore, integrating high-throughput genomics and transcriptomics technologies to analyze the spatiotemporal regulatory mechanisms of MORF proteins will help reveal how plants respond to environmental changes. This research offers new insights into plant adaptive evolution and provides potential applications for improving crop stress resistance and adaptability.

## 5. Conclusions

This study provides initial bioinformatics evidence of MORF protein family members in the fern Osmundaceae family, suggesting that ferns may share a conserved RNA editing regulatory module with angiosperms, as indicated by the presence of the conserved MORF-box domain in *Osmunda japonica* and *Plenasium vachellii*. Chloroplast editing frequencies (e.g., 0.7–1.0 in conserved sites) may be potentially influenced by the presence or number of MORF proteins, with *P. vachellii* (3 MORFs) showing slightly higher frequencies than *O. japonica* (1 MORF), though no direct correlation is implied. However, *P. banksiifolium*, where no MORF proteins were identified due to lower-depth NCBI SRA data, exhibited comparable chloroplast editing patterns, suggesting that core RNA editing machinery or other factors may maintain baseline editing levels, pending deeper sequencing. Core editing sites (shared across tissues) showed near-complete editing (frequency ≈1.0), while tissue-specific sites had lower editing levels (0.1–0.2), suggesting MORF proteins may contribute to editing events associated with basal metabolism, though this requires experimental validation. Comparative analysis of organelle RNA editing across species further revealed positive and negative correlations between distinct amino acid substitution types, indicating the presence of editing synergy and antagonism, and suggesting potential competition among editing events. Notably, Osmundaceae species showed a strong preference for Ser→Leu and Pro→Leu substitutions (>60% of total edits). These substitutions may enhance hydrophobic core stability, potentially aiding adaptation to environmental stress in ferns. Collectively, our findings provide preliminary evidence of MORF-mediated RNA editing in early vascular ferns, offering initial insights into organellar gene regulation. These observations may inform future studies on plant stress resistance (e.g., via targeted editing factors in breeding programs), though further experimental studies are needed to validate MORF roles and explore their broader implications across plant groups.

## Figures and Tables

**Table 1:** Physicochemical characterization of the MORF proteins in *A. thaliana*, *O. japonica* and *P. vachellii*.

Protein ID	Molecular Weight	Theoretical pI	Number of Amino Acids	Instability Index	Aliphatic Index	Grand Average of Hydropathicity
MORF1	45,157.41	7.65	419	56.54	44.06	−0.891
MORF2	24,714.83	8.52	219	54.46	64.98	−0.700
MORF3	27,558.30	9.02	244	67.46	62.37	−0.625
MORF4	78,822.18	8.47	721	44.76	43.29	−1.001
MORF5	26,024.51	9.21	229	51.15	66.86	−0.669
MORF6	26,367.98	9.08	232	52.03	64.27	−0.601
MORF7	21,622.74	9.35	188	55.85	79.79	−0.538
MORF8	42,869.70	9.38	395	66.48	47.95	−0.949
MORF9	26,173.49	8.99	232	56.06	64.31	−0.709
OsMORF9	24,818.96	8.90	229	56.89	58.60	−0.476
OsMORF3	25,140.50	9.16	228	62.26	71.54	−0.486
OsMORF2a	24,668.83	9.21	223	65.48	63.90	−0.661
OsMORF2b	19,720.09	5.64	172	61.97	60.06	−0.897
OsMORF2c	24,690.81	9.24	223	63.14	62.20	−0.709
OsMORF8a	43,298.76	8.65	398	62.28	47.41	−0.949
OsMORF8b	42,933.10	8.35	396	61.12	47.40	−0.998
OJ_27007	21,169.77	8.40	191	58.30	58.32	−0.684
PV_23919	53,930.48	6.87	495	60.53	39.11	−1.079
PV_35689	26,111.66	9.55	231	61.90	62.51	−0.672
PV_33438	43,009.38	9.31	393	63.51	48.96	−0.898

## Data Availability

Raw data have been deposited to National Center for Biotechnology Information (NCBI) under the BioProject number PRJNA1321311 (review link: https://dataview.ncbi.nlm.nih.gov/object/PRJNA1321311?reviewer=5f7f7vkep2bibb9df8qe18an40, accessed on 9 September 2025).

## Supplementary Materials

The following supporting information can be downloaded at https://www.mdpi.com/article/10.3390/biology14101463/s1, Supplementary Table S1: Chloroplast-encoded genes of *O. japonica* and *P. vachellii*. Supplementary Figure S1: Sampling Photographs of *Osmunda japonica*, *Plenasium banksiifolium* and *Plenasium vachellii*. Supplementary Figure S2: UpSet plot illustrating the intersection counts of RNA editing sites among the six tissue samples. Technique details for Chloroplast Genome Sequencing of *Osmunda japonica* and *Plenasium vachellii*.

## References

[1] Gott J.M., Emeson R.B. (2000). Functions and mechanisms of RNA editing. Annu. Rev. Genet..

[2] Benne R., Van den Burg J., Brakenhoff J.P., Sloof P., Van Boom J.H., Tromp M.C. (1986). Major transcript of the frameshifted coxII gene from trypanosome mitochondria contains four nucleotides that are not encoded in the DNA. Cell.

[3] Ichinose M., Sugita M. (2016). RNA Editing and Its Molecular Mechanism in Plant Organelles. Genes.

[4] Hao W., Liu G., Wang W., Shen W., Zhao Y., Sun J., Yang Q., Zhang Y., Fan W., Pei S. (2021). RNA Editing and Its Roles in Plant Organelles. Front. Genet..

[5] Covello P.S., Gray M.W. (1989). RNA editing in plant mitochondria. Nature.

[6] Hoch B., Maier R.M., Appel K., Igloi G.L., Kossel H. (1991). Editing of a chloroplast mRNA by creation of an initiation codon. Nature.

[7] Gao C., Li T., Zhao X., Wu C., Zhang Q., Zhao X., Wu M., Lian Y., Li Z. (2023). Comparative analysis of the chloroplast genomes of Rosa species and RNA editing analysis. BMC Plant Biol..

[8] Wang Y., Tan B.C. (2025). Pentatricopeptide repeat proteins in plants: Cellular functions, action mechanisms, and potential applications. Plant Commun..

[9] Shi X., Bentolila S., Hanson M.R. (2016). Organelle RNA recognition motif-containing (ORRM) proteins are plastid and mitochondrial editing factors in Arabidopsis. Plant Signal. Behav..

[10] Boyd R.D., Hayes M.L. (2023). A ribonuclease activity linked to DYW1 in vitro is inhibited by RIP/MORF proteins. Sci. Rep..

[11] Barkan A., Small I. (2014). Pentatricopeptide repeat proteins in plants. Annu. Rev. Plant Biol..

[12] Wang Y., Li H., Huang Z.Q., Ma B., Yang Y.Z., Xiu Z.H., Wang L., Tan B.C. (2023). Maize PPR-E proteins mediate RNA C-to-U editing in mitochondria by recruiting the trans deaminase PCW1. Plant Cell.

[13] Zeng Y., Dong J., Fu D., Shi M., Zheng Z., Zhong M., Wang H.B., Duan S.J., Jin H.L. (2024). The HPE1 RNA-binding protein modulates chloroplast RNA editing to promote photosynthesis under cold stress in Arabidopsis. FEBS Lett..

[14] Takenaka M., Zehrmann A., Verbitskiy D., Kugelmann M., Hartel B., Brennicke A. (2012). Multiple organellar RNA editing factor (MORF) family proteins are required for RNA editing in mitochondria and plastids of plants. Proc. Natl. Acad. Sci. USA.

[15] Zhang Q., Shen L., Ren D., Hu J., Chen G., Zhu L., Gao Z., Zhang G., Guo L., Zeng D. (2019). Characterization, Expression, and Interaction Analyses of OsMORF Gene Family in Rice. Genes.

[16] Wang D., Meng S., Su W., Bao Y., Lu Y., Yin W., Liu C., Xia X. (2019). Genome-Wide Analysis of Multiple Organellar RNA Editing Factor Family in Poplar Reveals Evolution and Roles in Drought Stress. Int. J. Mol. Sci..

[17] Xiong Y., Fang J., Jiang X., Wang T., Liu K., Peng H., Zhang X., Zhang A. (2022). Genome-Wide Analysis of Multiple Organellar RNA Editing Factor (MORF) Family in Kiwifruit (*Actinidia chinensis*) Reveals Its Roles in Chloroplast RNA Editing and Pathogens Stress. Plants.

[18] Xing J., Zhang Y., Song W., Ali N.A., Su K., Sun X., Sun Y., Jiang Y., Zhao X. (2024). Comprehensive identification, characterization, and expression analysis of the MORF gene family in *Brassica napus*. BMC Plant Biol..

[19] Yan J., Zhang Q., Guan Z., Wang Q., Li L., Ruan F., Lin R., Zou T., Yin P. (2017). MORF9 increases the RNA-binding activity of PLS-type pentatricopeptide repeat protein in plastid RNA editing. Nat. Plants.

[20] Wu J., Wang Y., Chen H., Xu T., Yang W., Fang X. (2025). Solid-like condensation of MORF8 inhibits RNA editing under heat stress in Arabidopsis. Nat. Commun..

[21] Ali N.A., Song W., Huang J., Wu D., Zhao X. (2024). Recent advances and biotechnological applications of RNA metabolism in plant chloroplasts and mitochondria. Crit. Rev. Biotechnol..

[22] Li J., Yuan J., Jing Y., Lin R. (2025). MORF proteins: A small family regulating organellar RNA editing and beyond. J. Integr. Plant Biol..

[23] Shim H., Lee H.J., Lee J., Lee H.O., Kim J.H., Yang T.J., Kim N.S. (2021). Plastid Genomes of the Early Vascular Plant Genus Selaginella Have Unusual Direct Repeat Structures and Drastically Reduced Gene Numbers. Int. J. Mol. Sci..

[24] Huang Y., Xing Y., Men W., Xue H., Hou W., Huang Y., Dou D., Kang T., Yang Y., Xu L. (2025). The first complete mitochondrial genome assembly and comparative analysis of the fern *Blechnaceae* family: *Blechnopsis orientalis*. Front. Plant Sci..

[25] Jin J.J., Yu W.B., Yang J.B., Song Y., dePamphilis C.W., Yi T.S., Li D.Z. (2020). GetOrganelle: A fast and versatile toolkit for accurate de novo assembly of organelle genomes. Genome Biol..

[26] Shi L., Chen H., Jiang M., Wang L., Wu X., Huang L., Liu C. (2019). CPGAVAS2, an integrated plastome sequence annotator and analyzer. Nucleic Acids Res..

[27] Gao F., Zhang C.T. (2006). GC-Profile: A web-based tool for visualizing and analyzing the variation of GC content in genomic sequences. Nucleic Acids Res..

[28] Greiner S., Lehwark P., Bock R. (2019). OrganellarGenomeDRAW (OGDRAW) version 1.3.1: Expanded toolkit for the graphical visualization of organellar genomes. Nucleic Acids Res..

[29] Lowe T.M., Eddy S.R. (1997). tRNAscan-SE: A program for improved detection of transfer RNA genes in genomic sequence. Nucleic Acids Res..

[30] Chen Y., Ye W., Zhang Y., Xu Y. (2015). High speed BLASTN: An accelerated MegaBLAST search tool. Nucleic Acids Res..

[31] Lewis S.E., Searle S.M., Harris N., Gibson M., Lyer V., Richter J., Wiel C., Bayraktaroglu L., Birney E., Crosby M.A. (2002). Apollo: A sequence annotation editor. Genome Biol..

[32] Potter S.C., Luciani A., Eddy S.R., Park Y., Lopez R., Finn R.D. (2018). HMMER web server: 2018 update. Nucleic Acids Res..

[33] Bailey T.L., Johnson J., Grant C.E., Noble W.S. (2015). The MEME Suite. Nucleic Acids Res..

[34] Bailey T.L., Boden M., Buske F.A., Frith M., Grant C.E., Clementi L., Ren J., Li W.W., Noble W.S. (2009). MEME SUITE: Tools for motif discovery and searching. Nucleic Acids Res..

[35] Grabherr M.G., Haas B.J., Yassour M., Levin J.Z., Thompson D.A., Amit I., Adiconis X., Fan L., Raychowdhury R., Zeng Q. (2011). Full-length transcriptome assembly from RNA-Seq data without a reference genome. Nat. Biotechnol..

[36] Li W., Godzik A. (2006). Cd-hit: A fast program for clustering and comparing large sets of protein or nucleotide sequences. Bioinformatics.

[37] Haas B.J., Papanicolaou A., Yassour M., Grabherr M., Blood P.D., Bowden J., Couger M.B., Eccles D., Li B., Lieber M. (2013). De novo transcript sequence reconstruction from RNA-seq using the Trinity platform for reference generation and analysis. Nat. Protoc..

[38] Tamura K., Stecher G., Kumar S. (2021). MEGA11: Molecular Evolutionary Genetics Analysis Version 11. Mol. Biol. Evol..

[39] Picardi E., Pesole G. (2013). REDItools: High-throughput RNA editing detection made easy. Bioinformatics.

[40] Li H. (2011). A statistical framework for SNP calling, mutation discovery, association mapping and population genetical parameter estimation from sequencing data. Bioinformatics.

[41] R Core Team R: A Language and Environment for Statistical Computing. https://www.R-project.org/.

[42] Knoop V. (2011). When you can’t trust the DNA: RNA editing changes transcript sequences. Cell. Mol. Life Sci..

[43] Chen M., Xia L., Tan X., Gao S., Wang S., Li M., Zhang Y., Xu T., Cheng Y., Chu Y. (2024). Seeing the unseen in characterizing RNA editome during rice endosperm development. Commun. Biol..

[44] Nie Y., Li Y., Yuan P., Wu C., Wang X., Wang C., Xu X., Shen Z., Hu Z. (2024). Arabidopsis Pentatricopeptide Repeat Protein GEND2 Participates in Mitochondrial RNA Editing. Plant Cell Physiol..

[45] Schuettpelz E., Schneider H., Smith A.R., Hovenkamp P., Prado J., Rouhan G., Salino A., Sundue M., Almeida T.E., Parris B. (2016). A community-derived classification for extant lycophytes and ferns. J. Syst. Evol..

[46] Fujii S., Small I. (2011). The evolution of RNA editing and pentatricopeptide repeat genes. New Phytol..

[47] Fauskee B.D., Sigel E.M., Pryer K.M., Grusz A.L. (2021). Variation in frequency of plastid RNA editing within Adiantum implies rapid evolution in fern plastomes. Am. J. Bot..

[48] Small I.D., Schallenberg-Rudinger M., Takenaka M., Mireau H., Ostersetzer-Biran O. (2020). Plant organellar RNA editing: What 30 years of research has revealed. Plant J..

[49] Rivals E., Bruyere C., Toffano-Nioche C., Lecharny A. (2006). Formation of the Arabidopsis pentatricopeptide repeat family. Plant Physiol..

[50] Liu R., Cao S.K., Sayyed A., Yang H.H., Zhao J., Wang X., Jia R.X., Sun F., Tan B.C. (2020). The DYW-subgroup pentatricopeptide repeat protein PPR27 interacts with ZmMORF1 to facilitate mitochondrial RNA editing and seed development in maize. J. Exp. Bot..

[51] Yang Y., Fan G., Zhao Y., Wen Q., Wu P., Meng Y., Shan W. (2020). Cytidine-to-Uridine RNA Editing Factor NbMORF8 Negatively Regulates Plant Immunity to Phytophthora Pathogens. Plant Physiol..

[52] Liang H., Deng J., Wang Y., Gao G., Yang R. (2025). The first complete mitochondrial genome of *Curcuma amarissima* (Zingiberaceae): Insights into multi-branch structure, codon usage, and phylogenetic evolution. BMC Genom..

[53] Wagoner J.A., Sun T., Lin L., Hanson M.R. (2015). Cytidine deaminase motifs within the DYW domain of two pentatricopeptide repeat-containing proteins are required for site-specific chloroplast RNA editing. J. Biol. Chem..

[54] Zhang K., Qu G., Zhang Y., Liu J. (2024). Assembly and comparative analysis of the first complete mitochondrial genome of *Astragalus membranaceus* (Fisch.) Bunge: An invaluable traditional Chinese medicine. BMC Plant Biol..

[55] Qi Z., Lu P., Long X., Cao X., Wu M., Xin K., Xue T., Gao X., Huang Y., Wang Q. (2024). Adaptive advantages of restorative RNA editing in fungi for resolving survival-reproduction trade-offs. Sci. Adv..

