# Supplementary material for: First Identification of MORF Family in Ferns: Molecular Regulation of Organellar RNA Editing in Osmunda japonica and Plenasium vachellii
Source: Biology (Basel). 2025 Oct 21;14(10):1463. doi: 10.3390/biology14101463 (PMC12561531; doi:10.3390/biology14101463)
Supplement: Supplementary file 1 [file biology-14-01463-s001.zip › biology-3899835-supplementary.pdf]

Supplementary Table1: Chloroplast-encoded genes of *Osmunda japonica* and *Plenasium vachellii.*

Group of genes	Name of genes
Subunits of NADH-dehydrogenase	*ndh*A, *ndh*B, *ndh*C, *ndh*D, *ndh*E, *ndh*F, *ndh*G, *ndh*H, *ndh*I, *ndh*J, *ndh*K
Subunits of photosystem Ⅰ	*psa*A, *psa*B, *psa*C, *psa*I, *psa*J, *psa*M
Subunits of photosystem Ⅱ	*psb*A, *psb*B, *psb*C, *psb*D, *psb*E, *psb*F, *psb*H, *psb*I, *psb*J, *psb*K, *psb*L, *psb*M, *psb*N, *psb*T, *psb*Z, *ycf*3
Subunits of cytochrome b/f complex	*pet*A, *pet*B, *pet*D, *pet*G, *pet*L, *pet*N
Subunits of ATP synthase	*atp*A, *atp*B, *atp*E, *atp*F, *atp*H, *atp*I
Large subunit of rubisco	*rbc*L
Small subunit of ribosome	*rps*2, *rps*3, *rps*4, *rps*7, *rps*8, *rps*11, *rps*12, *rps*14, *rps*15, *rps*16, *rps*18, *rps*19
Large subunit of ribosome	*rpl*2, *rpl*14, *rpl*16, *rpl*20, *rpl*21, *rpl*22, *rpl*23, *rpl*32, *rpl*33, *rpl*36
DNA dependent RNA polymerase	*rpo*A, *rpo*B, *rpoC*1, *rpoC*2
rRNA genes	*rrn*4.5S(×2), *rrn*5S(×2), *rrn*16S(×2), *rrn*23S(×2)
tRNA genes	*trn*A-UGC(×2), *trn*C-GCA, *trn*D-GUC, *trn*E-UUC, *trn*F-GAA, *trnf*M-CAU, *trn*G-GCC, *trn*G-UCC, *trn*H-GUG, *trn*I-CAU, *trnI* GAU(×2), *trn*K-CUU, *trn*L-CAA, *trn*L-UAA, *trn*L-UAG, *trn*M-CAU, *trn*N-GUU(×2), *trn*P-GGG, *trn*P-UGG, *trn*Q-UUG, *trn*R-ACG(×2), *trn*R-CCG, *trn*R-UCU, *trn*S-GCA, *trn*S-GCU, *trn*S-GGA, *trn*S-UGA, *trn*T-GGU, *trn*T-UAU, *trn*V-GAC(×2), *trn*V-UAC, *trn*W-CCA, *trn*Y-GUA
Maturase	*mat*K
c-type cytochrome synthesis gene	*ccs*A
Envelope membrane protein	*cem*A
Protease	*clp*P
Subunit of Acetyl-CoA-carboxylase	*accD*
Translational initiation factor	*inf*A
Protochlorophyllide reductase	*chl*B, *chl*L, *chl*N
Genes of unknown functions Open Reading	*ycf*1, *ycf*2, *ycf*4, *ycf*12

[1] The numbers in parentheses indicate the copy number of the gene, e.g., (×2) represents two copies.

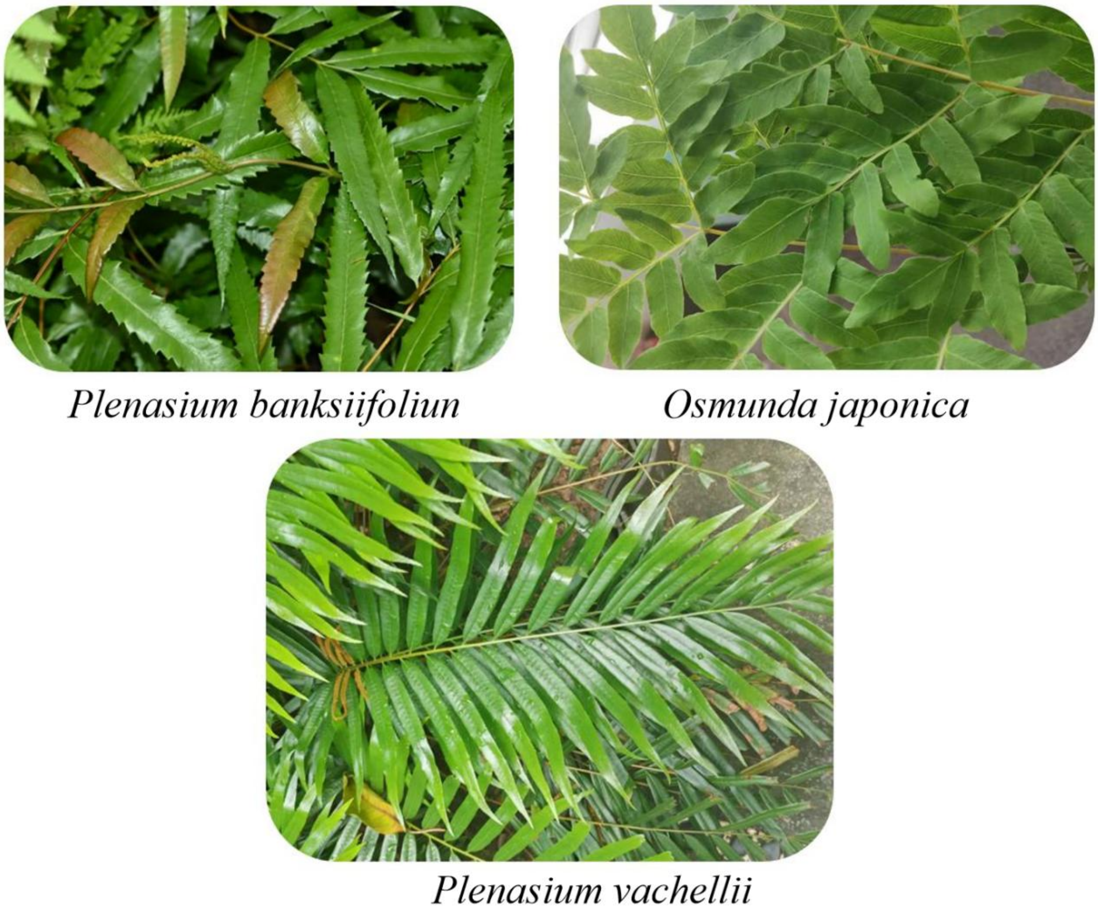

Supplementary Figure S1: Sampling Photographs of *Osmunda japonica*, *Plenasium banksiifolium* and *Plenasium vachellii*.

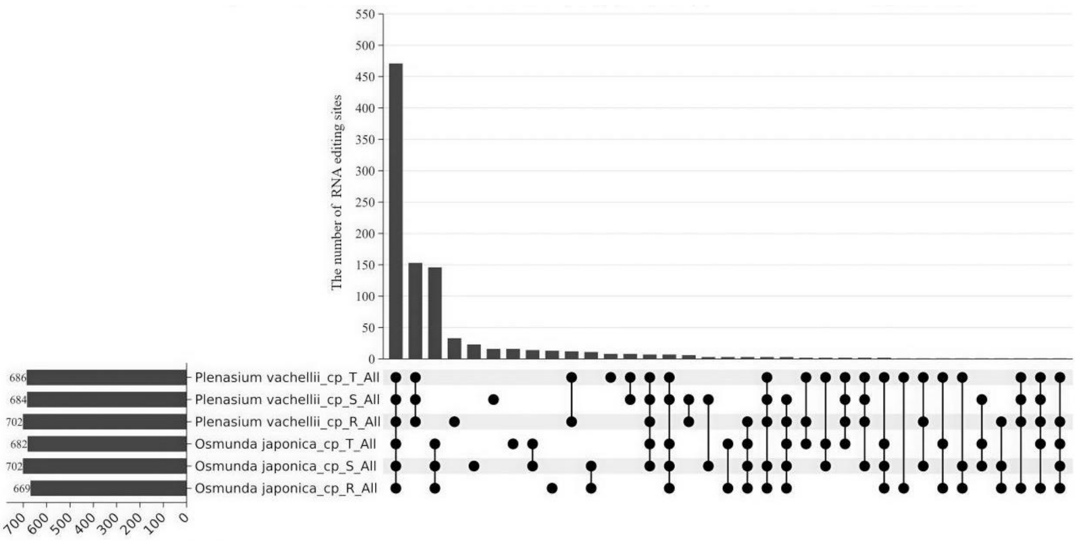

Supplementary Figure S2: UpSet plot illustrating the intersection counts of RNA editing sites among the six tissue samples.

Technique details for Chloroplast Genome Sequencing of *Osmunda japonica* and *Plenasium vachellii*

Total chloroplast DNA was extracted from the samples using a plant genomic DNA extraction kit (TIANGEN). After the total DNA samples were verified as qualified by Wuhan Benagen Technology Co., Ltd., paired-end sequencing was performed on the Illumina HiSeq 2500 sequencing platform. The obtained raw data were converted into raw sequencing sequences (Raw Reads) through CASAVA base calling analysis. Sequences containing adapters and low-quality sequences were filtered out to obtain clean data (Clean Data) for subsequent analysis.

Data processing was conducted using Trimmomatic v0.32 software (Bolger et al., 2014), following these steps:

(1) Removal of sequences containing N bases;

(2) Removal of adapter sequences from the reads;

(3) Trimming of low-quality bases (Q-value < 20) from the 3' to 5' direction of the reads;

(4) Trimming of low-quality bases (Q-value < 20) from the 5' to 3' direction of the reads;

(5) Trimming of reads where the average base quality of every four consecutive bases was less than 20;

(6) Removal of reads shorter than 50 nt and their paired reads.

The clean data were assembled using Velvet v1.2.03 software (Zerbino et al., 2008).
